# Multilingual profiles and their association with cognition and brain structure among older Indian adults

**DOI:** 10.1002/alz70857_099414

**Published:** 2025-12-24

**Authors:** Miguel Arce Renteria, Iris Strangmann, Yingxu Liu, Pranali Khobragade, Sarah Petrosyan, Niranjan Khandelwal, Joyita Banerjee, Emma Nichols, Erik Meijer, Shrikanth Narayanan, Kirsten M Lynch, Leon M. Aksman, Jinkook Lee

**Affiliations:** ^1^ Department of Neurology, Columbia University Medical Center, New York City, NY, USA; ^2^ Columbia Unversity Medical Center, New York, NY, USA; ^3^ Mark and Mary Stevens Neuroimaging and Informatics Institute, University of Southern California, Los Angeles, CA, USA; ^4^ Center for Economic and Social Research, University of Southern California, Los Angeles, CA, USA; ^5^ University of Southern California, Los Angeles, CA, USA; ^6^ National Institute for Medical Sciences and Research Centre, Jaipur, Rajasthan, India; ^7^ All India Institute of Medical Sciences, New Delhi, India

## Abstract

**Background:**

Multilingualism might confer a cognitive advantage and prevent pathological cognitive aging; however, it is unclear what aspect of multilingualism drives observed associations. Multilingual adults have heterogenous linguistic characteristics such as in age of second language (L2) acquisition (AoA), degree of L2 proficiency, and frequency of dual language use. Leveraging the Longitudinal Aging Study in India–Diagnostic Assessment of Dementia (LASI‐DAD), we evaluated whether active multilingual adults with combined early AoA, high L2 proficiency, and daily dual language use demonstrated better cognitive and neuroimaging outcomes compared to multilingual adults with passive linguistic characteristics.

**Method:**

The analytic sample included 492 multilingual older adults (*M*age[*SD*]=71[6.4], *M*years of education[*SD*]=7.1[5.6], 27% without any formal schooling, 50% female). Participants completed a language history questionnaire and a comprehensive cognitive battery assessing the domains of executive functioning, language, and memory. A subsample of participants (*n* = 69) completed 3T MRI scanning. Participants were classified into: Active multilinguals (*n* = 105, early AoA, high proficiency, and daily dual language use); passive multilinguals (*n* = 66, late AoA, low proficiency, and less than daily dual language use); and two mixed groups with either high (*n* = 216) or low (*n* = 105) proficiency but varied AoA and daily use. All models adjusted for relevant sociodemographic factors.

**Result:**

Active multilinguals demonstrated better executive functioning (B=0.22,[0.05,0.39]) and language performance (B=0.23,[0.03,0.43]) than passive multilinguals. The high proficient mixed group had better executive functioning (B=0.31,[0.15,0.47]) than passive multilinguals. There were no differences between the groups in terms of memory, total gray matter, hippocampal volume, or cortical thickness (all *p*'s > .05). Comparing associations between brain structure and cognition across multilingual groups, the association of hippocampal volume with executive functioning (B=0.26,[0.00,0.50]) and memory (B=0.24,[0.00,0.40]) was stronger among the passive groups than among the active group (B=‐0.11,[‐0.50,0.30] executive functioning, B=‐0.00,[‐0.50,0.50] memory).

**Conclusion:**

Among linguistically and socioeconomically diverse older multilingual Indian adults, active multilingualism (i.e., early AoA, high L2 proficiency, and daily dual language use) was associated with better cognitive functioning. Active multilingualism might also confer cognitive reserve given the weakened associations between hippocampal volume and cognition. However, future studies with larger neuroimaging sample sizes and longitudinal follow‐up are required to further evaluate brain‐cognition relationships among aging multilingual adults.

